# Locus Coeruleus Malfunction Is Linked to Psychopathology in Prodromal Dementia With Lewy Bodies

**DOI:** 10.3389/fnagi.2021.641101

**Published:** 2021-03-01

**Authors:** Niels Hansen

**Affiliations:** Department of Psychiatry and Psychotherapy, University Medical Center Goettingen, Göttingen, Germany

**Keywords:** locus coeruleus, noradrenaline, neurodegeneration, psychiatry, prodromal dementia with Lewy bodies

## Abstract

**Background:** The locus coeruleus (LC) is a nucleus in the human brainstem with a variety of noradrenaline-driven functions involved in cognition, emotions, and perception. Dementia with Lewy bodies (DLB) constitutes a neurodegenerative disease involving deposits of alpha-synuclein, first appearing in the brainstem. The goal of this narrative review is to delineate the relationship between the expression of psychiatric symptoms as an early-onset of DLB and the degeneration of the LC's noradrenaline system.

**Methods:** We searched in PubMed for relevant articles concerning LC degeneration and psychiatric symptoms in prodromal DLB in this narrative review. We rely on the McKeith criteria for prodromal psychiatric DLB.

**Results:** We found four studies that document neuronal loss, deposits of Lewy bodies and other hints for neurodegeneration in the LC in patients with DLB. Furthermore, we reviewed theories and studies on how the degenerated noradrenaline LC system contributes to psychiatric DLB's phenotype. We hypothesized how anxiety, hallucinations, delusions, and depressive symptoms might occur in DLB patients due to degenerated noradrenergic neurons entailing consecutive altered noradrenergic transmission in the LC's projection areas.

**Conclusions:** LC degeneration in prodromal DLB might cause psychiatric symptoms as the first and non-motor manifestation of DLB, as the LC is affected earlier by degeneration than are dopaminergic structures such as the substantia nigra, which are impaired later in the disease course.

## Introduction

The locus coeruleus (LC) is the small, bilaterally shaped structure in the brainstem located in the pons that contributes to cognitive functions (Berridge and Waterhouse, 2003; Sara and Bouret, 2012; Grueschow et al., 2020; James et al., 2020; Poe et al., 2020). The LC is the main location of a highly flexible, adaptable noradrenergic system projecting in many cortical and subcortical brain regions, thus explaining its highly relevant function in humans in everyday life, and essential for us humans as feeling and thinking beings. Beside its involvement in cognitive processes, a broader function recently emerged covering aspects of psychopathology, such as triggering anxiety (Morris et al., 2020), psychosis in alpha-synucleinopathies (Wolters, 2001) and demotivation in schizophrenia (Mäki-Marttunen et al., 2020), inattention to visual stimuli in the attention deficit-hyperactivity and autism-spectrum disorders (Boxhoorn et al., 2020), depressive symptoms in Alzheimer's disease (AD) (Förstl et al., 1992), suicide behavior (Roy et al., 2017) and defective emotional-memory encoding (Jacobs et al., 2020).

**Figure 1:**
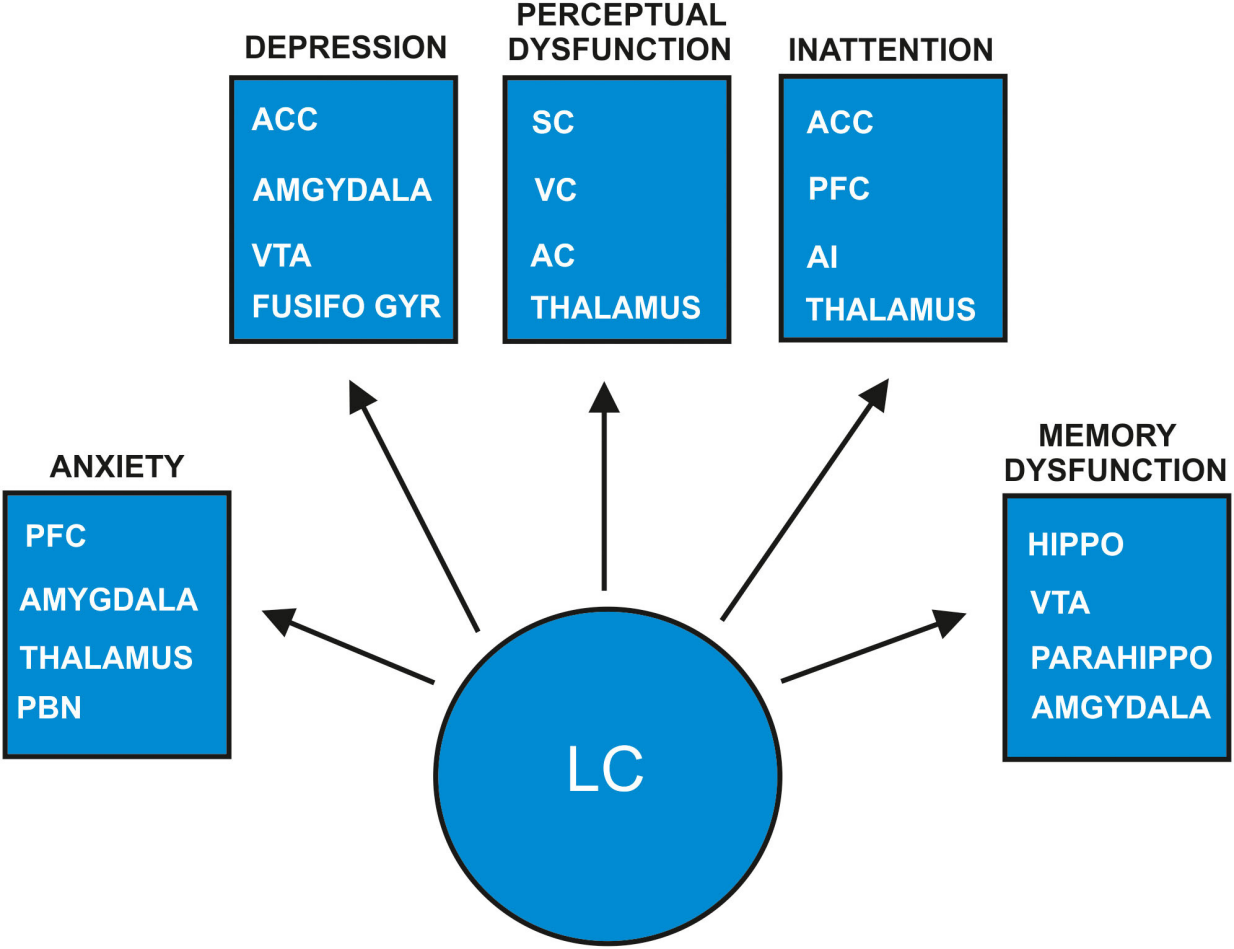
Proposed model of how psychiatric symptoms develop in prodromal dementia with Lewy bodies due to locus-coeruleus degeneration. The arrows depict the projection areas in the locus coeruleus (LC) noradrenaline system are suspected to be involved in the genesis of psychiatric symptoms as anxiety, depression, perceptual dysfunction, inattention and memory dysfunction. The indicated specific neuronal pathways for each psychopathologic category such as anxiety, depression, perceptual dysfunction, inattention and memory dysfunction are supposed to be degenerated due to early LC affection in prodromal DLB patients. AC, auditory cortex; ACC, anterior cingulate cortex; AI, anterior insula; Fusifo Gyr, fusiform gyrus; Parahippo, parahippocampal gyrus; PBN, parabrachial nucleus; PFC, prefrontal cortex; SC, somatosensory cortex; VC, visual cortex; VTA, ventral tegmental area.

These studies imply the involvement of LC activity in generating psychiatric symptoms such as fear, delusions, hallucinations, and inattention. The conditions and environmental factors inducing psychopathologic symptoms are manifold in diverse disease manifestations that conclusions regarding the development of psychiatric disease due to impaired noradrenergic LC activity cannot be drawn. As mentioned earlier, the LC's network is extremely important, and if degenerated, provides a link to its potential brain dysfunctions. The LC's widespread projections to the prefrontal cortex, limbic system containing structures such as the hippocampus, and amygdala and well as other cortical and subcortical regions such as the sensory cortex (Schwarz and Luo, 2015; Liebe et al., 2020) are crucial in generating psychiatric symptoms (Figure 1). These pathways are being intensively investigated, and novel pathways have recently been identified. Attention control, for instance, is mainly regulated by two different but relevant coeruleo-frontal cortical pathways recently disentangled by Bari's group (Bari et al., 2020). Moreover, both projections from the LC to cortical regions and back-projections are fundamental, such as the connection from the prefrontal cortex to the LC for LC-activity guided attentional behavior, as recently demonstrated in a study by Kuo et al. (2020). Recent evidence indicates dynamic interplay between the amygdala and hippocampus in emotional memory-processing guided by arousal-driven LC activity (Jacobs et al., 2020), which can be compromised by LC dysfunction. Furthermore, an extensive grid of noradrenergic projections to cortical regions including the somatosensory cortex involving interrelationships with other sensory systems are relevant for perception; thus disorders of perception might be affected by modulating noradrenergic LC activity (Figure 1) (McBurney-Lin et al., 2019). Taken together, the noradrenergic LC system seems to be a modulator of various cognitive and emotional states that are susceptible to a damaged noradrenergic LC system.

## Psychiatric Prodromal Lewy Body Dementia

Dementia with Lewy bodies (DLB) is a disorder hallmarked by a demential syndrome and underlying alpha-synucleinopathy with Lewy bodies. Several clinical core criteria must be fulfilled to diagnose DLB, ranging from symptoms of Parkinsonism, fluctuating attention and cognition, rapid eye movement (REM) sleep behavioral disorder, and visual hallucinations according to the McKeith criteria (McKeith et al., 2017). However, psychiatric symptoms such as anxiety, delusions, or hallucinations other than visual ones are considered only supporting criteria that do not yet justify a DLB diagnosis in patients not presenting core features apart from the indicative biomarker such as a nigrostriatal deficit in (123)-I-2-ß-carbomethoxy-3ß-(4-iodophenyl)-N-(3-fluoropropyl) nortropane SPECT (123I-FP-CIT SPECT). The term prodromal DLB with psychiatric features was recently coined as research criteria referring to a prodromal stage of DLB in which psychiatric symptoms predominate (McKeith et al., 2020). The assessment of DLB's typical core features can be confounded by psychiatric symptoms, thereby hindering a bradykinesia assessment or neuropsychological testing. Recent evidence suggests that the frequent detection of nigrostriatal deficit via 123I-FP-CIT SPECT as an indicative biomarker for DLB in patients presenting late-onset depression (Kazmi et al., 2020) might support the hypothesis that DLB begins as a late-onset psychiatric disorder. Diverse studies provided evidence that psychiatric symptoms such as acoustic hallucinations, delusions, and depressive symptoms at disease onset are much more often present in DLB than in AD patients in clinically diagnosed DLB and autopsy-confirmed DLB patients (Ballard et al., 1999). Furthermore, a psychiatric onset of DLB was discerned in nearly half of 234 DLB patients (Utsumi et al., 2020), thus supporting psychiatric prodromal DLB as a main clinical entity of prodromal DLB.

## Degeneration of the Locus Coeruleus in Dementia With Lewy Bodies

The LC reveals degeneration and a loss of noradrenergic cells in alpha-synucleinopathies such as DLB (Iseki et al., 2001; Mori et al., 2002; Brunnström et al., 2011; Haglund et al., 2016, Table 1). The noradrenergic LC system often becomes degenerated early in both alpha-synucleinopathies such as Parkinson's disease (PD) (Solopchuk et al., 2018; Li et al., 2019), and ß-amyloidopathies like Alzheimer's disease (AD) (Zarow et al., 2003; Hou et al., 2021). Both diseases are closely related to Lewy body pathology, as mixed pathologies can coexist in patients with cognitive impairment (Coughlin et al., 2020). Lewy body dementia includes the terms PD dementia and DLB (Haider et al., 2020). Although our review focusses on prodromal DLB, it is important to report on insights into LC degeneration in PD patients with cognitive impairment, as the diseases are sometimes difficult to distinguish. For example, the exact time at which motor or cognitive symptoms begin is often unclear. The pathomechanism might be similar in both alpha-synucleinopathies at an early stage in terms of the amount, type, and spread of Lewy bodies within the LC region. *Via* neuromelanin-sensitive MRI, a recent study (Li et al., 2019) showed that the LC's contrast-to-noise ratio in 23 PD patients with mild cognitive impairment was lower than in control subjects. They observed a negative relationship between reduced contrast to the LC and measures of executive dysfunction in these patients (Li et al., 2019) implying an early contribution by the LC– noradrenaline system to symptomatology in PD patients. Another interesting study (Solopchuk et al., 2018) in patients with PD assessed how specific symptoms were associated with LC and subtantia nigra (SN) degeneration. They found a correlation between the LC's neuromelanin-imaging values and depression (Solopchuk et al., 2018). Taking a similar approach would thus be optimal to disentangle depressive symptoms in patients with prodromal LBD to probe for the relationship between early affective symptoms in DLB and LC dysfunction. A recent study (Fischer et al., 2021) investigated the neurodegeneration within the LC and SN and correlation between the observed degeneration in these nuclei and the occurrence of psychiatric symptoms such as anxiety, depression, and psychosis. Surprisingly, they found no association in PD patients between the aforementioned symptoms and neuronal loss and gliosis in the LC, but in the SN. This investigation points toward a major role played by the SN in the development of psychopathology in PD patients. However, this psychopathological development might differ between PD patients presenting neuronal degeneration in the SN and prodromal DLB patients. Possible reasons for that are that (i) the LC is already affected early in DLB patients, and (ii) psychopathological symptoms might occur due to noradrenergic LC dysfunction.

**Table 1:** Studies involving degeneration of locus coeruleus—noradrenaline system in patients with dementia with Lewy bodies.

**Number of Subjects**	**Mean age in years**	**Results effects of LC degeneration**	**References**
*N* = 26 DLB/PDD	79	DLB/PDD and AD greater LC damage vs. FTLD, VaD	Haglund et al., 2016
*N* = 25 DLB/PDD	75	DLB/PDD and AD higher LC degeneration vs. FTLD, VaD	Brunnström et al., 2011
*N* = 4 LB PSP	78	LB and neuronal loss in LC	Mori et al., 2002
*N* = 9 DLB	75	Neuronal loss in all DLB cases	Iseki et al., 2001

*AD, Alzheimer's disease dementia; DLB, dementia with Lewy bodies; FTLD, frontotemporal dementia; LB, Lewy bodies; LC, locus coeruleus; N, number; PDD, Parkinson's disease dementia; PSP, progressive supranuclear palsy; VaD, vascular disease dementia; vs, versus. The age is shown as mean in years*.

To our knowledge, no working group is currently addressing this issue. The degeneration in prodromal DLB starts early in the LC, and precedes the neurodegeneration within the SN (Del Tredici et al., 2002). This is why non-motoric features may predominate in DLB's prodromal stage. Furthermore, an animal model (Bjerkén et al., 2019) demonstrated that the early degeneration of LC neurons might exacerbate the dopaminergic neuronal loss in the SN and impair its functionality as depicted by striatal dopamine release. In other words, the LC degeneration in DLB might pave the way for later SN degeneration along with worsening psychopathology related to dopaminergic dysfunction. Hypocretin-neuronal loss in the LC in addition to noradrenergic cells also are part of this degeneration (Kasanuki et al., 2014), having potential consequences for sleep dysfunction by impairing arousal function. However, there have been no investigations to date addressing neurodegeneration in prodromal DLB. It would also be worth knowing how LC degeneration in prodromal DLB relates to neuroinflammation, which seems to have an impact on LC degeneration in alpha-synucleinopathies [as recently reviewed by Giorgi et al. (2020) and shown in animal models (Wang et al., 2020)].

## Noradrenergic Mechanisms Inducing Psychiatric Symptoms in Prodromal Dementia With Lewy Bodies

The loss of noradrenergic cells in the LC may lead to alterations in noradrenaline and its derivatives, thus impairing various brain functions. A main metabolite of noradrenaline called 3-methoxy-4-hydroxyphenylglycol (MHPG) was demonstrated to be higher in DLB than AD patients in cerebrospinal fluid, whereas peripheral blood level of MHPG was decreased in DLB compared to AD patients (Janssens et al., 2018). These findings suggest substantial alterations in noradrenaline and its metabolites especially in DLB patients, probably due to LC degeneration. We thus speculate that altered noradrenergic transmission in LC projection regions and LC terminals due to LC degeneration could affect psychiatric symptomatology in prodromal DLB with predominant non-motoric features (Figure 1 for an overview of noradrenergic LC function in relation to neurodegeneration due to DLB). Many animal studies employing diverse methodologies proved that anxiety is dependent on the noradrenergic LC-amygdala (McCall et al., 2015, 2017; Llorca-Torralba et al., 2019), the LC-parabrachial nucleus (PBN) (Yang et al., 2021) and the orexinergic LC-thalamus circuits (Heydendael et al., 2014). In addition, the damage to noradrenergic LC cells resulted in increased anxiety in addition to defective social capacities (Song et al., 2019) as well as depressive behavior (Itoi et al., 2011) in rodents. These studies suggest that LC degeneration entailing noradrenergic cell loss might enable anxiety in humans, although strong evidence from DLB patients and LC affection is lacking. For anxiety generation, an LC-amygdala and thalamus circuit is proposed in our model (Figure 1).

Concerning depressive symptoms, there is evidence that patients with depression reveal a reduced LC neuromelanin contrast signal (Shibata et al., 2007), indicative of LC degeneration. We know that mainly dopaminergic neuronal loss in the ventral tegmental area (VTA), but not LC is diagnosed in patients presenting symptoms of late-onset depression, and in post-mortem evidence of Lewy bodies (Wilson et al., 2013). These findings were recently corroborated by the fact that 24% of patients suffering from late-onset depression exhibited a nigrostriatal dopaminergic deficit in 123I-Ioflupane SPECT (Kazmi et al., 2020). However, the VTA is not the only source of dopamine, as it can also be released from LC terminals in the hippocampus (Kempadoo et al., 2016) and prefrontal cortex (Devoto et al., 2020) indicating that a neuronal LC loss could entail impaired dopamine release as a possible consequence. Depression due to LC degeneration might be thereby explained. Prodromal LBD patients often suffer delusions in addition to depressive symptoms. The development of depressive symptoms is probably associated with decreased LC activity mediated (1) by the LC amygdala pathway and (2) the interconnection between the VTA and LC and (3) the anterior cingulate cortex and fusiform gyrus (Del Cerro et al., 2020) (Figure 1). The development of delusions might be generated by combined abnormal degenerated noradrenergic structures resulting in altered dopaminergic neurotransmission, as the dopamine hypothesis is one theory explaining the induction of psychosis (Meltzer and Stahl, 1976). Concurring with this assumption, Wolters (2001) hypothesized further that the fluctuating decrease in noradrenergic LC activity combined with degenerated dopaminergic neurons in the SN in alpha-synucleinopathies might result in psychotic symptoms (Figure 1). In addition, the perception of salient stimuli is guided by LC activation, thus the generation of misperception as a possible psychotic symptom might be influenced by altered LC activity caused by degeneration in addition to the stimuli perception interacting and modulating with the LC's cortical projecting areas (Figure 1). Furthermore, attention deficits caused by impaired noradrenergic transmission in coeruleo-frontal pathways is a potential additional factor triggering visual hallucinations (Figure 1), similar to the phenomenon of visual hallucinations in DLB patients presenting frontal, basal ganglia and insula atrophy measured via voxel-based morphometry, and attention deficits, as Pezzoli recently proposed (Pezzoli et al., 2019). Understanding how the LC-noradrenaline system works in conjunction with the genesis of sensory, visual or auditory experiences is relevant for the genesis of tactile, visual, or acoustic hallucinations in prodromal DLB patients. The LC can activate “silent” neurons after processing sensory stimuli called sensory gating (Devilbiss and Waterhouse, 2004)—important for processing novel sensory stimuli, whose function can be lost through LC degeneration in DLB. In addition, the LC's noradrenaline system enables the modulation of task-specific sensory signals within the somatosensory cortex (for review see Waterhouse and Navarra, 2019) that is potentially affected by noradrenergic degeneration, leading to dysfunctional perception. Noradrenergic LC projections lead to an inhibition of neuronal responses in the visual cortex (McLean and Waterhouse, 1994; Stowell et al., 2019) and in the auditory cortex (Salgado et al., 2012). In particular, finely tuned synaptic responses in the auditory cortex in the rat depended on the LC's firing mode determined by the arousal level (Shinba et al., 2000). The LC system's modulation enabling these specific inhibitory connections to the auditory and visual cortices might be absent in DLB patients' degenerated LC (Figure 1). The abnormal perceptions of DLB patients might be due to the cortical hyperexcitability observed in human visual and auditory cortices when abnormal perception is present (Spencer et al., 2009; Braithwaite et al., 2013, 2015). To summarize: these findings above suggest that in humans, degeneration of the LC noradrenaline system in prodromal DLB patients might induce neuronal hyperexcitability in the visual and auditory cortices due to reduced inhibition via LC projections (Figure 1). Another aspect to be aware of is that the LC noradrenaline system is believed to optimize neural gain (Aston-Jones and Cohen, 2005)—assessable by measuring the diameter of pupil dilation in humans. Impaired noradrenergic transmission caused by LC degeneration in prodromal DLB patients might explain the reduced neural gain in these patients. Noradrenergic dysfunction might in turn result in sensory-prediction errors triggering the inability to reduce incoming stimuli due to their prior predictions, and finally the incapacity to put the sensory experience in a context, a condition already incorporated in the “disconnection hypothesis” in the development of schizophrenic symptoms (Friston et al., 2016). Compromised LC function in prodromal DLB patients thus seems to be responsible for the genesis of their delusional perception and hallucinations (Figure 1). Another characteristic of DLB patients is fluctuating attentional dysfunction, which might result from impaired noradrenergic transmission within the coerulo-frontal pathway (Figure 1). Moreover, noradrenaline helps to ensure the function of selective attention (Dahl et al., 2020). The crosstalk between the frontoparietal and salience network comprising structures such as the anterior cingulated cortex and anterior insula are important for LC-driven attentional control (Lee et al., 2020). Therefore, a dysfunctional LC system might explain the dysfunctional attention control in prodromal DLB patients, as LC projections to the frontal and anterior cingulated cortices, as well as the anterior insula might receive less activation due to neuronal LC loss (Figure 1).

There is another prodromal DLB subtype besides psychiatric prodromal DLB that is characterized by mild cognitive impairment (McKeith et al., 2020). Episodic memory dysfunction exists in DLB, and might be related to the spread of alpha synuclein pathology in hippocampal subfields CA2/3 (Coughlin et al., 2020) as relevant structures for memory formation. Furthermore, Coughlin et al. (2020) demonstrated that hippocampal tau protein within the hippocampus subregion CA2/3 correlated negatively with memory function in patients with LBD. Given the LC's pivotal role in memory processing at various memory stages (Sara, 2015; Hansen, 2017) as shown in animal models (Hansen and Manahan-Vaughan, 2015a,b) and humans (Jacobs et al., 2015, 2020) it is likely that LC degeneration with its LC terminals affect hippocampal memory-processing in prodromal DLB patients. There is evidence from neuroimaging studies in AD patients that LC degeneration affects functional connectivity between memory-relevant structures such as the parahippocampus and the LC (Jacobs et al., 2015) leading to memory dysfunction. It is hypothesized that memory functions are impaired in patients with AD due to LC degeneration entailing consecutive dysfunction of cellular correlates of long-term memory formation (James et al., 2020). Animal models have revealed the LC's relevant role in hippocampal long-term plasticity in different hippocampal subregions such as the dentate gyrus and CA1 region (Lemon et al., 2009; Hansen and Manahan-Vaughan, 2015a,b). Interaction between the frontal cortex and hippocampus is essential for memory formation (Eichenbaum, 2017). As the LC projects to both regions with afferent fibers (Schwarz and Luo, 2015), it is tempting to postulate that LC degeneration might affect both neuronal pathways concurrently, thus leading to memory dysfunction in prodromal DLB patients (Figure 1).

Taken together, a hypothesis is provided for LC involvement in generating psychiatric symptoms such as depression, anxiety, and psychosis including delusions and hallucinations (Figure 1) that may characterize psychiatric-onset prodromal DLB. Future research should aim to determine established and novel genetic, molecular, and imaging biomarkers associated with the dopaminergic and especially the noradrenergic LC system and their interplay in prodromal DLB patients, thereby optimizing diagnostics and specific treatment for such patients.

## Synopsis

Taken together, the LC noradrenaline system is a promising nucleus that should be targeted in future neuroimaging studies in prodromal DLB patients. Furthermore, investigations focusing on noradrenaline biomarkers in blood and cerebrospinal fluid should guide researchers to discover the relationship between the noradrenergic system's *in vivo* biomarkers and the psychopathology of prodromal DLB patients. Taking this approach, we believe it will be possible to optimize diagnostic tools for detecting prodromal DLB.

## Perspective

Considered together, we provide a framework for the postulated relationship between early-appearing psychopathology and LC dysfunction due to Lewy body pathology in prodromal DLB patients. Future research should address the potential relationship between psychopathology and LC degeneration along with LC dysfunction in prodromal DLB patients. *In vivo* neuromelanin-sensitive imaging of the LC seems to be an adequate tool for this purpose, as it is known to be sensitive in neurodegenerative diseases such as PD or AD (Betts et al., 2019). In addition, studies addressing functional connectivity *via* fMRI would be of merit in prodromal DLB patients to assess the LC system's functionality with its various connections relevant for LC function in prodromal DLB patients. LC neuroimaging is sensitive enough to depict the LC's integrity, as Hämmerer et al. (2018) showed in elderly subjects—a method that might also apply in prodromal DLB patients. *Via in vivo* LC imaging, it would be possible to stratify patients such as prodromal DLB patients in clinical trials according to their noradrenergic dysfunction. Furthermore, we want to apply monoaminergic and molecular biomarkers to assess LC system degeneration that might be linked to neuropsychiatric symptoms in prodromal DLB [as is evident in patients with AD (Jacobs et al., 2019)]. Noradrenaline-enhancing drugs such as noradrenaline reuptake inhibitors could be used in further trials to demonstrate the role and extent of LC system involvement in prodromal LBD patients. More investigation is warranted to explore the usefulness of noradrenergic transmission-enhancing drugs as a therapeutic target in prodromal DLB, as discussed recently (Vermeiren and De Deyn, 2017). Keeping in mind that LC degeneration even precedes SN degeneration and could worsen dopaminergic SN degeneration, it would seem worthwhile to administer noradrenaline-reuptake inhibitors to prevent further brain damage in prodromal DLB. Noradrenaline-enhancing treatment should be investigated in future studies with large cohorts of prodromal DLB patients. The stratification of prodromal DLB patients via LC imaging would help us identify suitable patients for such a treatment regimen that could be implemented later, after large-scale studies have delivered evidence on its safety, tolerability, and efficacy in novel treatment guidelines.

## Author Contributions

The author confirms being the sole contributor of this work and has approved it for publication.

## Conflict of Interest

The author declares that the research was conducted in the absence of any commercial or financial relationships that could be construed as a potential conflict of interest.

## References

[B1] Aston-Jones G. Cohen J. D. (2005). An integrative theory of locus coeruleus-norepinephrine function: adaptive gain and optimal performance. Annu. Rev. Neurosci. 28, 403–450. 10.1146/annurev.neuro.28.061604.135709 16022602

[B2] Ballard C. G. Shaw F. Lowery K. McKeith I. Kenny R. (1999). The prevalence, assessment and associations of falls in dementia with Lewy bodies and Alzheimer's disease. Dement. Geriatr. Cogn. Disord. 10, 97–103. 10.1159/000017108 10026382

[B3] Bari A. Xu S. Pignatelli M. Takeuchi D. Feng J. Li Y. (2020). Differential attentional control mechanisms by two distinct noradrenergic coeruleo-frontal cortical pathways. Proc. Natl. Acad. Sci. U.S.A. 117, 29080–29089. 10.1073/pnas.2015635117 33139568 PMC7682591

[B4] Berridge B. W. Waterhouse B. D. (2003). The locus coeruleus-noradrenergic system: modulation of behavioral state and state-dependent cognitive processes. Brain Res. Brain Res. Rev. 42, 33–84. 10.1016/s0165-0173(03)00143-7 12668290

[B5] Betts M. J. Kirilina E. Otaduy M. C. G. Ivanov D. Acosta-Cabronero J. Callaghan M. F.. (2019). Locus coeruleus imaging as a biomarker for noradrenergic dysfunction in neurodegenerative diseases. Brain 142, 2558–2571. 10.1093/brain/awz193 31327002 PMC6736046

[B6] Bjerkén S. A. Persson R. S. Barkander A. Karalija N. Pelegrina-Hidalgo N. Gerhardt G. A.. (2019). Noradrenaline is crucial for the substantia nigra dopaminergic cell maintenance. Neurochem. Int. 131:104551. 10.1016/j.neuint.2019.104551 31542295

[B7] Boxhoorn S. Bast N. Supèr H. Polzer L. Cholemkery H. Freitag C. M. (2020). Pupil dilation during visuospatial orienting differentiates between autism spectrum disorder and attention-deficit/hyperactivity disorder. J. Child Psychol. Psychiatry 61, 614–624. 10.1111/jcpp.13179 31853987

[B8] Braithwaite J. J. Broglia E. Bagshaw A. P. Wilkins A. J. (2013). Evidence for elevated cortical hyperexcitability and its association with out-of-body experiences in the non-clinical population: new findings from a pattern-glare task. Cortex 49, 793–805. 10.1016/j.cortex.2011.11.013 22209090

[B9] Braithwaite J. J. Mevorach C. Takahashi C. (2015). Cortex. stimulating the aberrant brain: evidence for increased cortical hyperexcitability from a transcranial direct current stimulation (tDCS) study of individuals predisposed to anomalous perceptions. Cortex 69, 1–13. 10.1016/j.cortex.2015.03.023 25967083

[B10] Brunnström H. Friberg N. Lindberg E. (2011). Englund differential degeneration of the locus coeruleus in dementia subtypes. Clin. Neuropathol. 30, 104–110. 10.5414/npp30104 21545773

[B11] Coughlin D. G. Ittyerah R. Peterson C. Phillips J. S. Miller S. Rascovsky K.. (2020). Hippocampal subfield pathologic burden in Lewy body diseases vs. Alzheimers Dis. Neuropathol. Appl. Neurobiol. 46, 707–721. 10.1111/nan.12659 PMC7787184 32892355

[B12] Dahl M. J. Mather M. Sander M. C. Werkle-Bergner M. (2020). Noradrenergic responsiveness supports selective attention across the adult lifespan. J. Neurosci. 40, 4372–4390. 10.1523/JNEUROSCI.0398-19.2020 32317388 PMC7252473

[B13] Del Cerro I. Martínez-Zalacaín I. Guinea-Izquierdo A. Gascón-Bayarri J. Viñas-Diez V. Urretavizcaya M.. (2020). Locus coeruleus connectivity alterations in late-life major depressive disorder during a visual oddball task. Neuroimage. Clin. 28:102482. 10.1016/j.nicl.2020.102482 33371943 PMC7649653

[B14] Del Tredici K. Rüb U. De Vos R. A. I. Bohl J. R. E. Braak H. (2002). Where does parkinson disease pathology begin in the brain? J. Neuropathol. Exp. Neurol. 61, 413–426. 10.1093/jnen/61.5.413 12030260

[B15] Devilbiss D. M. Waterhouse B. D. (2004). The effects of tonic locus ceruleus output on sensory-evoked responses of ventral posterior medial thalamic and barrel field cortical neurons in the awake rat. J. Neurosci. 24, 10773–10785. 10.1523/JNEUROSCI.1573-04.2004 15574728 PMC6730210

[B16] Devoto P. Sagheddu C. Santoni M. Flore G. Saba P. Pistis M. (2020). Noradrenergic Source of dopamine assessed by microdialysis in the medial prefrontal cortex. Front. Pharmacol. 11:588160. 10.3389/fphar.2020.588160 33071798 PMC7538903

[B17] Eichenbaum H. (2017). Prefrontal-hippocampal interactions in episodic memory. Nat. Rev. Neurosci. 18, 547–558. 10.1038/nrn.2017.74 28655882

[B18] Fischer N. M. Hinkle J. T. Perepezko K. I. Bakker C. C. Morris M. Broen M. P. G.. (2021). Brainstem pathologies correlate with depression and psychosis in parkinson's disease. Am. J. Geriatr. Psychiatry. 20, 30577–7. 10.1016/j.jagp.2020.12.009 33455856 PMC8277871

[B19] Förstl H. Burns A. Levy R. Cairns N. Luthert P. Lantos P. (1992). Neurologic signs in Alzheimer's disease. Results of a prospective clinical and neuropathologic study. Arch. Neurol. 49, 1038–1042. 10.1001/archneur.1992.00530340054018 1417511

[B20] Friston K. Brown H. R. Siemerkus J. Stephan K. E. (2016). The dysconnection hypothesis Schizophr. Res. 176, 83–94. 10.1016/j.schres.2016.07.014 27450778 PMC5147460

[B21] Giorgi F. S. Biagioni F. Galgani A. Pavese N. Lazzeri G. Fornai F. (2020). Locus Coeruleus modulates neuroinflammation in parkinsonism and dementia. Int. J. Mol. Sci. 21:8630. 10.3390/ijms21228630 33207731 PMC7697920

[B22] Grueschow M. Kleim B. Ruff C. C. (2020). Role of the locus coeruleus arousal system in cognitive control. J. Neuroendocrinol. 32:e12890. 10.1111/jne.12890 32820571

[B23] Haglund M. Friberg N. Danielsson E. J. D. Norrman J. Englund E. (2016). A methodological study of locus coeruleus degeneration in dementing disorders. Clin. Neuropathol. 35, 287–294. 10.5414/NP300930 27191912

[B24] Haider A. Spurling B. C. Sánchez-Manso J. C. (2020). Lewy Body Dementia in StatPearls [Internet]. Treasure Island, FL: StatPearls Publishing. 29494048

[B25] Hämmerer D. Callaghan M. F. Hopkins A. Kosciessa J. Betts M. Cardenas-Blanco A.. (2018). Locus coeruleus integrity in old age is selectively related to memories linked with salient negative events. Proc. Natl. Acad. Sci. U.S.A. 115, 2228–2233. 10.1073/pnas.1712268115 29440429 PMC5834676

[B26] Hansen N. (2017). The longevity of hippocampus-dependent memory is orchestrated by the locus coeruleus-noradrenergic system. Neural. Plast. 2017:2727602. 10.1155/2017/2727602 28695015 PMC5485371

[B27] Hansen N. Manahan-Vaughan D. (2015a). Hippocampal long-term potentiation that is elicited by perforant path stimulation or that occurs in conjunction with spatial learning is tightly controlled by beta-adrenoreceptors and the locus coeruleus. Hippocampus 25, 1285–1298. 10.1002/hipo.22436 25727388 PMC6680149

[B28] Hansen N. Manahan-Vaughan D. (2015b). Locus coeruleus stimulation facilitates long-term depression in the dentate gyrus that requires activation of beta-adrenergic receptors. Cereb. Cortex 25, 1889–1896. 10.1093/cercor/bht429 24464942 PMC4459289

[B29] Heydendael W. Sengupta A. Beck S. Bhatnagar S. (2014). Optogenetic examination identifies a context-specific role for orexins/hypocretins in anxiety-related behavior. Physiol. Behav. 130, 182–190. 10.1016/j.physbeh.2013.10.005 24140988 PMC4155939

[B30] Hou R. Beardmore R. Holmes C. Osmond C. Darekar A. (2021). A case-control study of the locus coeruleus degeneration in Alzheimer's disease. Eur. Neuropsychopharmacol. 43, 153–159. 10.1016/j.euroneuro.2020.12.013 33431221

[B31] Iseki E. Kato M. Marui W. Uéda K. Kosaka K. (2001). A neuropathological study of the disturbance of the nigro-amygdaloid connections in brains from patients with dementia with Lewy bodies. Neurol. Sci. 185, 129–134. 10.1016/s0022-510x(01)00481-6 11311294

[B32] Itoi K. Sugimoto N. Suzuki S. Sawada K. Das G. Uchida K.. (2011). Targeting of locus ceruleus noradrenergic neurons expressing human interleukin-2 receptor α-subunit in transgenic mice by a recombinant immunotoxin anti-Tac(Fv)-PE38: a study for exploring noradrenergic influence upon anxiety-like and depression-like behaviors. J. Neurosci. 31, 6132–6139. 10.1523/JNEUROSCI.5188-10.2011 21508238 PMC6632972

[B33] Jacobs H. I. Priovoulos N. Poser B. A. Pagen L. H. Ivanov D. Verhey F. R.. (2020). Dynamic behavior of the locus coeruleus during arousal-related memory processing in a multi-modal 7T fMRI paradigm. Elife 9:e52059. 10.7554/eLife.52059 32579109 PMC7343392

[B34] Jacobs H. I. Wiese S. van de Ven V. Gronenschild E. H. Verhey F. R. Matthews P. M. (2015). Relevance of parahippocampal-locus coeruleus connectivity to memory in early dementia. Neurobiol. Aging. 36, 618–626. 10.1016/j.neurobiolaging.2014.10.041 25433457

[B35] Jacobs H. I. L. Riphagen J. M. Ramakers I. H. G. B. Verhey F. R. J. (2019). Alzheimer'**s** disease pathology: pathways between central norepinephrine activity, memory, and neuropsychiatric symptoms. Mol. Psychiatry. [Epub ahead of print]. 10.1038/s41380-019-0437-x 31138892

[B36] James T. Kula B. Choi S. Khan S. S. Bekar L. K. Smith N. A. (2020). Locus coeruleus in memory formation and Alzheimer's disease. Eur. J. Neurosci. [Epub ahead of print]. 10.1111/ejn.15045 33190318 PMC8121900

[B37] Janssens J. Vermeiren Y. Fransen E. Aerts T. Van Dam D. Engelborghs S.. (2018). Cerebrospinal fluid and serum MHPG improve Alzheimer's disease versus dementia with Lewy bodies differential diagnosis. Alzheimers Dement. (Amst) 10, 172–181. 10.1016/j.dadm.2018.01.002 29552632 PMC5852321

[B38] Kasanuki K. Iseki E. Kondo D. Fujishiro H. Minegishi M. Sato K.. (2014). Neuropathological investigation of hypocretin expression in brains of dementia with Lewy bodies. Neurosci. Lett. 569, 68–73. 10.1016/j.neulet.2014.03.020 24704327

[B39] Kazmi H. Walker Z. Booij J. Khan F. Shah S. Sudre C. H.. (2020). Late onset depression: dopaminergic deficit and clinical features of prodromal Parkinson's disease: a cross-sectional study. J. Neurol. Neurosurg. Psychiatry 92, 158–164. 10.1136/jnnp-2020-324266 33268471 PMC7841491

[B40] Kempadoo K. A. Mosharov E. V. Choi S. J. Sulzer D. Kandel E. R. (2016). Dopamine release from the locus coeruleus to the dorsal hippocampus promotes spatial learning and memory. Proc. Natl. Acad. Sci. U.S.A. 113, 14835–14840. 10.1073/pnas.1616515114 27930324 PMC5187750

[B41] Kuo C. C. Hsieh J. C. Tsai H. C. Kuo Y. S. Yau H. J. Chen C.-C.. (2020). Inhibitory interneurons regulate phasic activity of noradrenergic neurons in the mouse locus coeruleus and functional implications. J. Physiol. 598, 4003–4029. 10.1113/JP279557 32598024

[B42] Lee T. H. Kim S. H. Katz B. Mather M. (2020). The decline in intrinsic connectivity between the salience network and locus coeruleus in older adults: implications for distractibility. Front. Aging Neurosci. 12:2. 10.3389/fnagi.2020.00002 32082136 PMC7004957

[B43] Lemon N. Aydin-Abidin S. Funke K. Manahan-Vaughan D. (2009). Locus coeruleus activation facilitates memory encoding and induces hippocampal LTD that depends on beta-adrenergic receptor activation. Cereb. Cortex. 19, 2827–2837. 10.1093/cercor/bhp065 19435710 PMC2774396

[B44] Li Y. Wang C. Wang J. Zhou Y. Ye F. Zhang Y.. (2019). Mild cognitive impairment in *de novo* Parkinson's disease: a neuromelanin MRI study in locus coeruleus. Mov. Disord. 34, 884–892. 10.1002/mds.27682 30938892

[B45] Liebe T. Kaufmann J. Meng L. Skalej M. Wagner G. Walter M. (2020). *In vivo* anatomical mapping of human locus coeruleus functional connectivity at 3 T MRI. Hum. Brain Mapp. 41, 2136–2151. 10.1002/hbm.24935 31994319 PMC7267980

[B46] Llorca-Torralba M. Suárez-Pereira I. Bravo L. Camarena-Delgado C. Garcia-Partida J. A. Mico J. A.. (2019). Chemogenetic silencing of the locus coeruleus-basolateral amygdala pathway abolishes pain-induced anxiety and enhanced aversive learning in rats. Biol. Psychiatry 15, 1021–1035. 10.1016/j.biopsych.2019.02.018 30987747

[B47] Mäki-Marttunen V. Andreassen O. A. Espeseth T. (2020). The role of norepinephrine in the pathophysiology of schizophrenia. Neurosci. Biobehav. Rev. 118, 298–314. 10.1016/j.neubiorev.2020.07.038 32768486

[B48] McBurney-Lin J. Lu J. Zuo Y. Yang H. (2019). Locus coeruleus-norepinephrine modulation of sensory processing and perception: a focused review. Neurosci. Biobehav. Rev. 105, 190–199. 10.1016/j.neubiorev.2019.06.009 31260703 PMC6742544

[B49] McCall J. G. Al-Hasani R. Siuda E. R. Hong D. Y. Norris A. J. Ford C. P. (2015). CRH Engagement of the locus coeruleus noradrenergic system mediates stress-induced anxiety. Neuron. 87, 605–620. 10.1016/j.neuron.2015.07.002 26212712 PMC4529361

[B50] McCall J. G. Siuda E. R. Bhatti D. L. Lawson L. A. McElligott Z. A. Stuber G. A.. (2017). Locus coeruleus to basolateral amygdala noradrenergic projections promote anxiety-like behavior. Elife 6:e18247. 10.7554/eLife.18247 28708061 PMC5550275

[B51] McKeith I. G. Boeve B. F. Dickson D. W. Halliday G. Taylor J. P. Weintraub D.. (2017). Diagnosis and management of dementia with Lewy bodies: fourth consensus report of the DLB consortium. Neurology 89, 88–100. 10.1212/WNL.0000000000004058 28592453 PMC5496518

[B52] McKeith I. G. Ferman T. J. Thomas A. J. Blanc F. Boeve B. F. Fujishiro H.. (2020). prodromal DLB diagnostic study group. Research criteria for the diagnosis of prodromal dementia with Lewy bodies. Neurology 94, 743–755. 10.1212/WNL.0000000000009323 32241955 PMC7274845

[B53] McLean J. Waterhouse B. D. (1994). Noradrenergic modulation of cat area 17 neuronal responses to moving visual stimuli. Brain Res. 667, 83–97. 10.1016/0006-8993(94)91716-7 7895086

[B54] Meltzer H. Y. Stahl S. M. (1976). The dopamine hypothesis of schizophrenia: a review. Schizophr. Bull. 2, 19–76. 10.1093/schbul/2.1.19 779020

[B55] Mori H. Oda M. Komori T. Arai N. Takanashi M. Mizutani T. (2002). Lewy bodies in progressive supranuclear palsy. Acta Neuropathol. 104, 273–278. 10.1007/s00401-002-0555-3 12172913

[B56] Morris L. S. McCall J. G. Charney D. S. Murrough J. W. (2020). The role of the locus coeruleus in the generation of pathological anxiety. Brain Neurosci. Adv. 10.1177/2398212820930321 32954002 PMC7479871

[B57] Pezzoli S. Cagnin A. Antonini A. Venneri A. (2019). Frontal and subcortical contribution to visual hallucinations in dementia with Lewy bodies and Parkinson's disease. Postgrad. Med. 131, 509–522. 10.1080/00325481.2019.1656515 31422718

[B58] Poe G. R. Foote S. Eschenko O. Johansen J. P. Bouret S. Aston-Jones G.. (2020). Locus coeruleus: a new look at the blue spot. Nat. Rev. Neurosci. 21, 644–659. 10.1038/s41583-020-0360-9 32943779 PMC8991985

[B59] Roy B. Wang Q. Palkovits M. Faludi G. Dwivedi Y. (2017). Altered miRNA expression network in locus coeruleus of depressed suicide subjects. Sci. Rep. 7:4387. 10.1038/s41598-017-04300-9 28663595 PMC5491496

[B60] Salgado H. Garcia-Oscos F. Martinolich L. Hall S. Restom R. Tseng K. Y.. (2012). Pre- and postsynaptic effects of norepinephrine on γ-aminobutyric acid-mediated synaptic transmission in layer 2/3 of the rat auditory cortex. Synapse 66, 20–28. 10.1002/syn.20979 21905124

[B61] Sara S. J. (2015). Locus coeruleus in time with the making of memories. Curr. Opin. Neurobiol. 35, 87–94. 10.1016/j.conb.2015.07.004 26241632

[B62] Sara S. J. Bouret S. (2012). Orienting and reorienting: the locus coeruleus mediates cognition through arousal. Neuron. 76, 130–141. 10.1016/j.neuron.2012.09.011 23040811

[B63] Schwarz L. A. Luo L. (2015). Organization of the locus coeruleus-norepinephrine system. Curr. Biol. 25, R1051–R1056. 10.1016/j.cub.2015.09.039 26528750

[B64] Shibata E. Sasaki M. Tohyama K. Otsuka K. Sakai A. (2007). Reduced signal of locus ceruleus in depression in quantitative neuromelanin magnetic resonance imaging. Neuroreport 18, 415–418. 10.1097/WNR.0b013e328058674a 17496795

[B65] Shinba T. Briois L. Sara S. J. (2000). Spontaneous and auditory-evoked activity of medial agranular cortex as a function of arousal state in the freely moving rat: interaction with locus coeruleus activity. Brain Res. 887, 293–300. 10.1016/s0006-8993(00)03009-2 11134618

[B66] Solopchuk O. Sebti M. Bouvy C. Benoit C. E. Warlop T. Jeanjean A.. (2018). Locus coeruleus atrophy doesn't relate to fatigue in Parkinson's disease. Sci. Rep. 8:12381. 10.1038/s41598-018-30128-y 30120287 PMC6098016

[B67] Song S. Wang Q. Jiang L. Oyarzabal E. Riddick N. V. Wilson B.. (2019). Noradrenergic dysfunction accelerates LPS-elicited inflammation-related ascending sequential neurodegeneration and deficits in non-motor/motor functions. Brain Behav. Immun. 81, 374–387. 10.1016/j.bbi.2019.06.034 31247288 PMC6754798

[B68] Spencer K. M. Niznikiewicz M. A. Nestor P. G. Shenton M. E. McCarley R. M. (2009). Left auditory cortex gamma synchronization and auditory hallucination symptoms in schizophrenia. BMC Neurosci. 10:85. 10.1186/1471-2202-10-85 19619324 PMC2719648

[B69] Stowell R. D. Sipe G. O. Dawes R. P. Hanna N. Batchelor H. N. Lordy K. A.. (2019). Noradrenergic signaling in the wakeful state inhibits microglial surveillance and synaptic plasticity in the mouse visual cortex. Nat. Neurosci. 22, 1782–1792. 10.1038/s41593-019-0514-0 31636451 PMC6875777

[B70] Utsumi K. Fukatsu R. Yamada R. Takamaru Y. Hara Y. (2020). Characteristics of initial symptoms and symptoms at diagnosis in probable dementia with Lewy body disease: incidence of symptoms and gender differences. Psychogeriatrics 20, 737–745. 10.1111/psyg.12586 32743894

[B71] Vermeiren Y. De Deyn P. P. (2017). Targeting the norepinephrinergic system in Parkinson's disease and related disorders: the locus coeruleus story. Neurochem. Int. 102, 22–32. 10.1016/j.neuint.2016.11.009 27899296

[B72] Wang Q. Oyarzabal E. A. Song S. Wilson B. Santos J. H. Hong J. S. (2020). Locus coeruleus neurons are most sensitive to chronic neuroinflammation-induced neurodegeneration. Brain Behav. Immun. 87, 359–368. 10.1016/j.bbi.2020.01.003 31923552 PMC7316605

[B73] Waterhouse B. D. Navarra R. L. (2019). The locus coeruleus-norepinephrine system and sensory signal processing: a historical review and current perspectives. Brain Res. 1709, 1–15. 10.1016/j.brainres.2018.08.032 30179606

[B74] Wilson R. S. Nag S. Boyle P. A. Hizel L. P. Yu L. Buchman A. S.. (2013). Brainstem aminergic nuclei and late-life depressive symptoms. JAMA Psychiatry 70, 1320–1328. 10.1001/jamapsychiatry.2013.2224 24132763 PMC3856195

[B75] Wolters E. C. (2001). Intrinsic and extrinsic psychosis in Parkinson's disease. J. Neurol. 248(Suppl. 3), 22–27. 10.1007/pl00007822 11697684

[B76] Yang B. Sanches-Padilla J. Kondapalli J. Morison S. L. Delpire E. Awatramani R.. (2021). Locus coeruleus anchors a trisynaptic circuit controlling fear-induced suppression of feeding. Neuron. 10.1016/j.neuron.2020.12.023 33476548 PMC9272546

[B77] Zarow C. Lyness S. A. Mortimer J. A. Chui H. C. (2003). Neuronal loss is greater in the locus coeruleus than nucleus basalis and substantia nigra in Alzheimer and Parkinson diseases. Arch. Neurol. 60, 337–341. 10.1001/archneur.60.3.337 12633144

